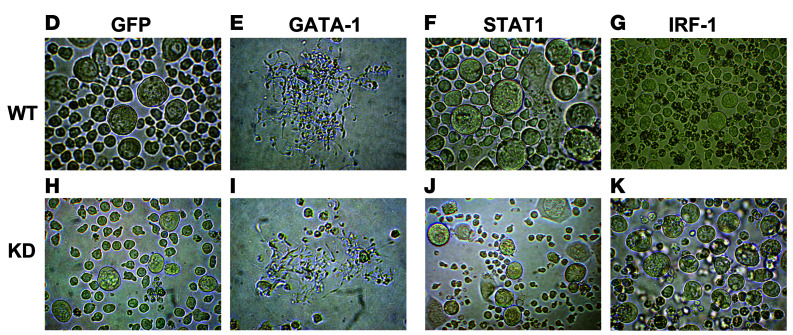# Corrigendum to STAT1 promotes megakaryopoiesis downstream of GATA-1 in mice

**DOI:** 10.1172/JCI195974

**Published:** 2025-07-15

**Authors:** Zan Huang, Terri D. Richmond, Andrew G. Muntean, Dwayne L. Barber, Mitchell J. Weiss, John D. Crispino

Original citation: *J Clin Invest*. 2007;117(12):3890–3899. https://doi.org/10.1172/JCI33010

Citation for this corrigendum: *J Clin Invest*. 2025;135(14):e195974. https://doi.org/10.1172/JCI195974

The authors recently became aware that Figure 6G in the original article was an inadvertent duplication of 6K. The correct Figure 6, D–K, provided from the original source data, is shown below. In addition, the authors were unable to retrieve the original source data for the STAT1 immunoblot in Figure 4C. An updated figure panel, derived from original source data from a replicate experiment, is provided below.

The authors regret the errors.

## Figures and Tables

**Figure 4C:**
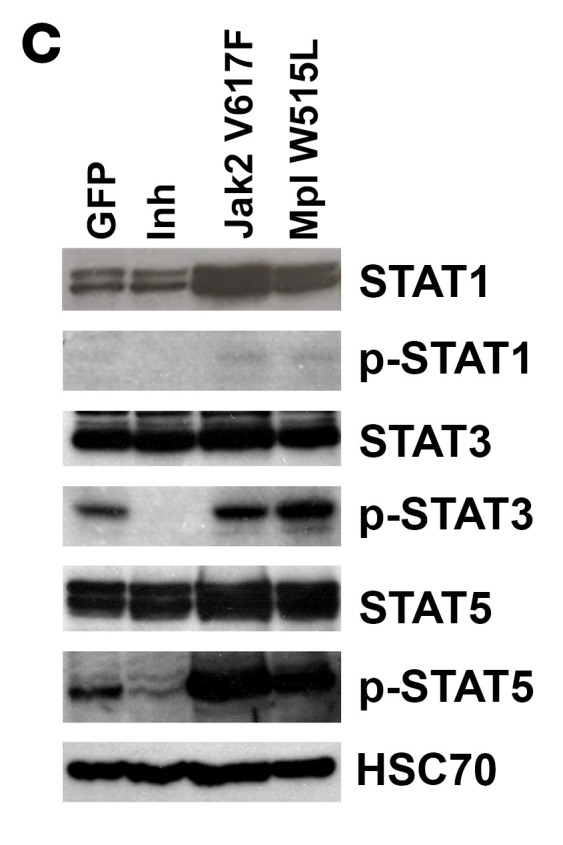


**Figure 6, D-K:**